# Heading Direction Is Significantly Biased by Preceding Whole-Body Roll-Orientation While Lying

**DOI:** 10.3389/fneur.2022.868144

**Published:** 2022-04-18

**Authors:** Alexander Andrea Tarnutzer, Vasco Duarte da Costa, Denise Baumann, Simone Hemm

**Affiliations:** ^1^Department of Neurology, Cantonal Hospital of Baden, Baden, Switzerland; ^2^Faculty of Medicine, University of Zurich, Zurich, Switzerland; ^3^School of Life Sciences, Institute for Medical Engineering and Medical Informatics, University of Applied Sciences and Arts Northwestern Switzerland, Muttenz, Switzerland

**Keywords:** prior knowledge, spatial orientation and navigation, post-tilt bias, perceived straight-ahead, inertial measurement unit, sensor shoe insoles

## Abstract

**Background:**

After a prolonged static whole-body roll-tilt, a significant bias of the internal estimates of the direction of gravity has been observed when assessing the subjective visual vertical.

**Objective:**

We hypothesized that this post-tilt bias represents a more general phenomenon, broadly affecting spatial orientation and navigation. Specifically, we predicted that after the prolonged roll-tilt to either side perceived straight-ahead would also be biased.

**Methods:**

Twenty-five healthy participants were asked to rest in three different lying positions (supine, right-ear-down, and left-ear-down) for 5 min (“adaptation period”) prior to walking straight-ahead blindfolded for 2 min. Walking was recorded with the inertial measurement unit sensors attached to different body locations and with sensor shoe insoles. The raw data was segmented with a gait–event detection method. The Heading direction was determined and linear mixed-effects models were used for statistical analyses.

**Results:**

A significant bias in heading into the direction of the previous roll-tilt position was observed in the post-adaptation trials. This bias was identified in both measurement systems and decreased again over the 2-min walking period.

**Conclusions:**

The bias observed further confirms the influence of prior knowledge on spatial orientation and navigation. Specifically, it underlines the broad impact of a shifting internal estimate of direction of gravity over a range of distinct paradigms, illustrating similar decay time constants. In the broader context, the observed bias in perceived straight-ahead emphasizes that getting up in the morning after a good night's sleep is a vulnerable period, with an increased risk of falls and fall-related injuries due to non-availability of optimally tuned internal estimates of the direction of gravity and the direction of straight-ahead.

## Introduction

For the spatial orientation and navigation in 3-dimensional space, accurate and precise estimates of self-motion and orientation relative to gravity are important. To keep track of the current body positions with reference to previous locations and surroundings (1, 2), input from several, partially redundant sensory systems are computationally combined within the central nervous system in a weighted fashion based on their reliability. Thereby, optimal internal estimates of the direction of gravity (3), self-motion (1, 4) and heading direction (5) are achieved.

Such awareness of one's spatial orientation and movement in the environment has been referred to as “spatial cognition” (6) and it is used to achieve a variety of goals, including navigation through space, maintaining postural control and identifying and acting on objects (2). Sensory input signals emerge from the peripheral–vestibular organs (measuring rotational and linear accelerations), the visual system and proprioception.

The perceptual estimates of the self-orientation relative to the gravity provide a straightforward means to quantify graviception at the level of the cortex. Due to their widespread availability and easy-to-understand instructions, visual line adjustments indicating the “subjective visual vertical” (SVV) are preferentially used to assess graviception. In behavioral studies investigating prolonged whole-body roll-tilt, concomitant drifts of the SVV (7, 8) and a bias (i.e., deviations of SVV) upon return to upright position (9), termed “post-tilt bias” were observed.

We have previously characterized this post-tilt bias using an SVV paradigm, demonstrating that it is usually toward the direction of previous whole-body roll-tilt (termed “adaptation position”) and that it decays exponentially [time constant = ~70 s (10)]. We favored the central mechanisms to explain the post-tilt bias and proposed a perceptual shift of perceived vertical toward the recent (roll-tilted) position based on prior knowledge (10). This concept describes a strategy relying on the assumption that an earth–vertical (upright) position is most likely and therefore assumes that the subject's recent whole-body orientation was approximately parallel to gravity. Accordingly, this prior knowledge is combined with sensory input in a Bayesian framework to estimate the most likely roll position (11–13). As a result, the perceived direction of gravity will be shifted toward the body-longitudinal axis. A similar effect was observed for vision-independent paradigms of verticality perception as the subjective haptic vertical, again showing significant post-tilt biases (14).

Internal estimates of the direction of gravity are not only important for verticality perception, but also for postural control and ambulation. Specifically, a significant post-tilt bias toward the adaptation position has been demonstrated for self-positioning in space relative to gravity after prolonged static roll tilt (15). In the patients with acute or persistent unilateral peripheral–vestibular deficits, both heading direction and self-alignment relative to straight-ahead have been shown to be biased toward the affected ear when removing vision. With eyes closed, strong ipsilesional walking deviations (16, 17) and ipsilesional whole-body rotational deviations when walking on the spot (18) have been observed. Furthermore, it has been shown that the repetitive one side predominant asymmetric (off-vertical axis) stimulation of the vestibular system influences the spatial representation of the subjective straight-ahead (SSA) and self-motion perception in the opposite direction of the most rapid stimulus (19, 20), emphasizing a significant otolithic component in this paradigm.

Thus, in analogy to the observed post-tilt bias in perceived direction of vertical, we hypothesized that after prolonged static roll-tilt a shift in the internal estimate of direction of gravity will bias heading direction during ambulation. Specifically, we predicted walking straight-ahead while blindfolded to be biased toward the adaptation side, resulting in a curved walking pattern. The magnitude of this effect is expected to decay exponentially. Alternatively, preserved straight-ahead walking would suggest that the post-tilt bias is a more restricted phenomenon, limited to verticality perception and self-positioning relative to gravity. To test this hypothesis, we measured walking with vision removed after prolonged static whole-body roll-tilt in different body positions relative to gravity in healthy human subjects using two different gait–assessment systems.

## Materials and Methods

### Ethical Approval

All subjects provided written informed consent after a full explanation of the experimental procedure. The study was approved by the Ethikkommission Nordwest- und Zentralschweiz (EKNZ, ID = 2020–01712) on the research involving humans. The research project was conducted in accordance with the university policies, the Federal Act on Data Protection, the Declaration of Helsinki (except for registration in a database), the principles of Good Clinical Practice, the Human Research Act (HRA) and the Human Research Ordinance (HRO). The data will be made available on request from the authors.

### Subjects

Twenty-five healthy, adult human subjects (9 females, 16 males, age [mean ± 1 standard deviation (SD): 29.4 ± 8.7 years, range, 20–60 years] were recruited for the study. The subjects weighed between 50 and 121 kg with an average height of 163.5 and 178.6 cm for female and male subjects, respectively. The vast majority of participating subjects reported that they were right-handed (22 of 25).

### Experimental Setup

**Figure 1:**
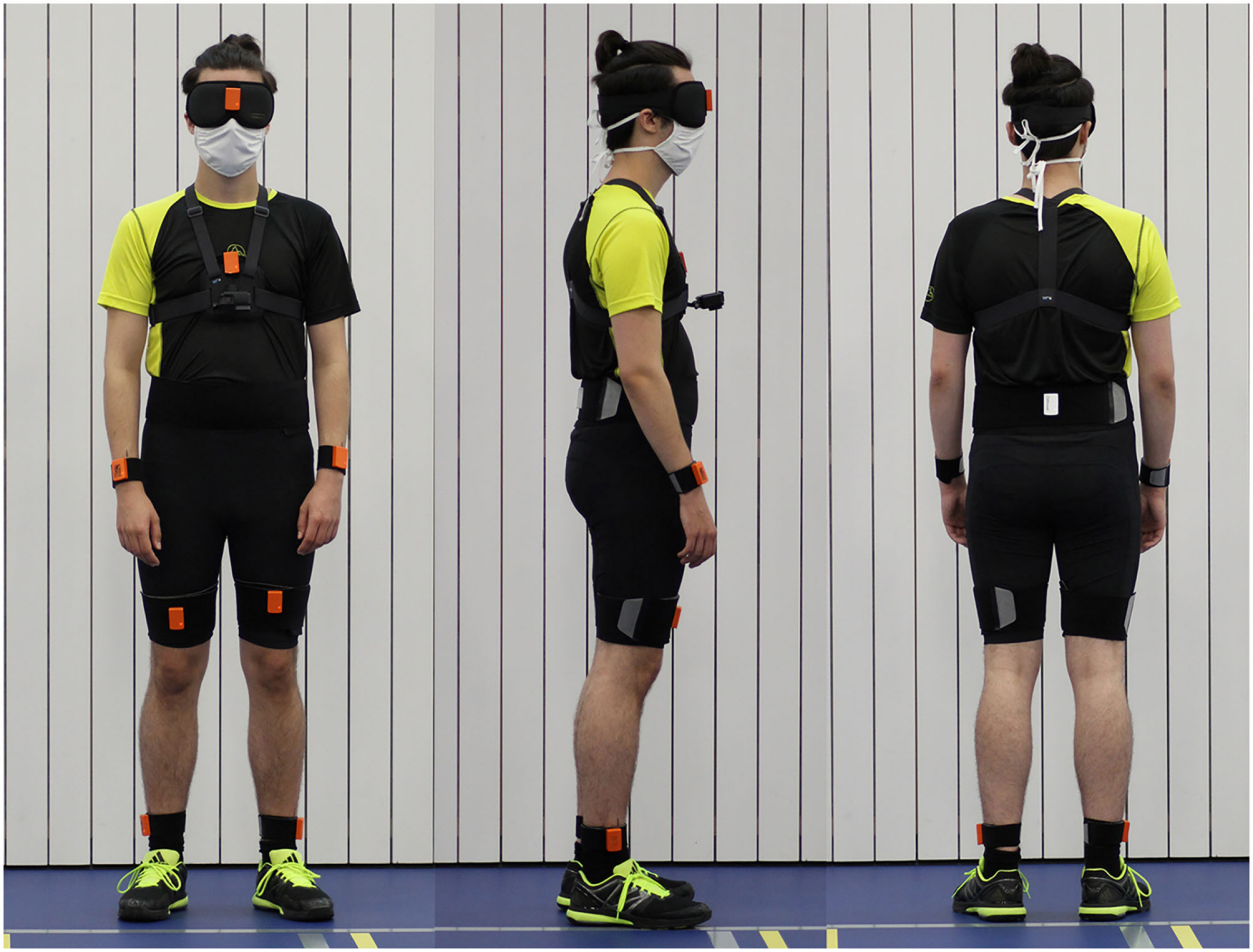
The fully-equipped participant during the study; 9 IMUs (MTw Awinda, Xsens Technologies B.V., Enschede, Netherlands) were attached to the subject with Velcro® patches and bands at both ankles, both thighs, lower back, chest, both wrists, and at the forehead. Sensory shoe insoles (Insole3, Moticon ReGo AG, Munich, Germany) were laid in the subject's own shoes. Additionally, the subject wore a GoPro Hero 6 on a chest mount (GoPro Inc., San Mateo CA, USA), a sleeping mask and earmuffs.

The trials were performed in a double sports hall (length, 31 m; width, 26 m). One of the sports hall's corners was selected as the starting point, where subjects could rest on two vaulting boxes before walking. All trials were performed while the participant was equipped as shown in Figure 1. The subjects had to wear a sleeping mask and earmuffs to eliminate visual cues during walking and to reduce auditory feedback for orientation in space, respectively.

### Sensor Systems

#### Inertial Measurement Unit (IMU) Based Motion Tracking

Motion was measured with nine inertial motion trackers (MTw Awinda, Xsens Technologies B.V., Enschede, Netherlands). This motion tracker is composed of a three-dimensional (3D) accelerometer, 3D gyroscope, 3D magnetometer, barometer, and a thermometer (21). Inertial motion trackers were placed at both ankles, both thighs, lower back, chest, both wrists, and at the forehead with Velcro® patches and bands, resulting in a total of nine sensors attached. The individual sensors were placed at the same defined body parts for every participant. The forehead sensor was directly placed onto the sleeping mask at the height of the nasion. The chest sensor was either positioned on a chest mount (Chesty, GoPro Inc., San Mateo CA, USA) or on a Velcro® band, which was wrapped around the subject's chest. Signals were recorded at a sampling rate of 100 Hz. Before each individual measurement, the sensor orientation of all motion trackers was reset while the subjects stood in neutral pose.

#### Inertial Pressure Measurement Sensory Shoe Insoles

Foot pressure was measured using sensory shoe insoles (Insole3, Moticon ReGo AG, Munich, Germany). The Insole3 is a thin, wireless measuring insole with an integrated 16 MB flash storage. It contains 16 pressure sensors and 1 IMU with a 3D-accelerometer and a 3D-gyroscope. The area covered by pressure sensors in relation to the total area of the sensor insoles is between 62.5 and 67.4%, with a higher sensor coverage for larger insoles. The origin is defined as the center of the shoe insole. Consequently, center of pressure measurements during normal walking is subject dependent and can vary from zero. The posterior–anterior (PA) direction is set as the positive *x*-direction and the lateral–medial (LM) direction as the positive *y*-direction thus resulting in an opposite orientation between the left and right foots. The shoe insoles were laid in the subject's own shoes. Before all measurements, the soles' pressure sensors were zeroed. Apart from pressure measurement data, the sensory shoe insoles also recorded data of an integrated inertial measurement unit sensor. The sensor insole data was recorded with a sample rate of 100 Hz.

Subjects were able to get used to the attached sensors by walking once with their eyes open and once with their eyes covered before data collection started. This acclimation phase lasted approximately 3 min to adjust the temperature of the sensory insoles to that of the shoe to reduce possible sensor drifts (22, 23). For later determination of eventual irregularities, the feet and floor were also filmed using a GoPro Hero 6 camera (GoPro Inc., San Mateo CA, USA).

### Experimental Paradigm

The baseline measurements were recorded first. Therefore, the subjects remained in a sitting–upright position for 1 min before walking (“baseline trials”). Subsequently, the test trials were collected. Each test trial consisted of an *adaptation phase* in different resting (i.e., whole-body horizontal) positions and a *walking phase* directly following the adaptation phase (for description, see the following paragraphs). For each resting position, three runs per participant were performed.

**Figure 2:**
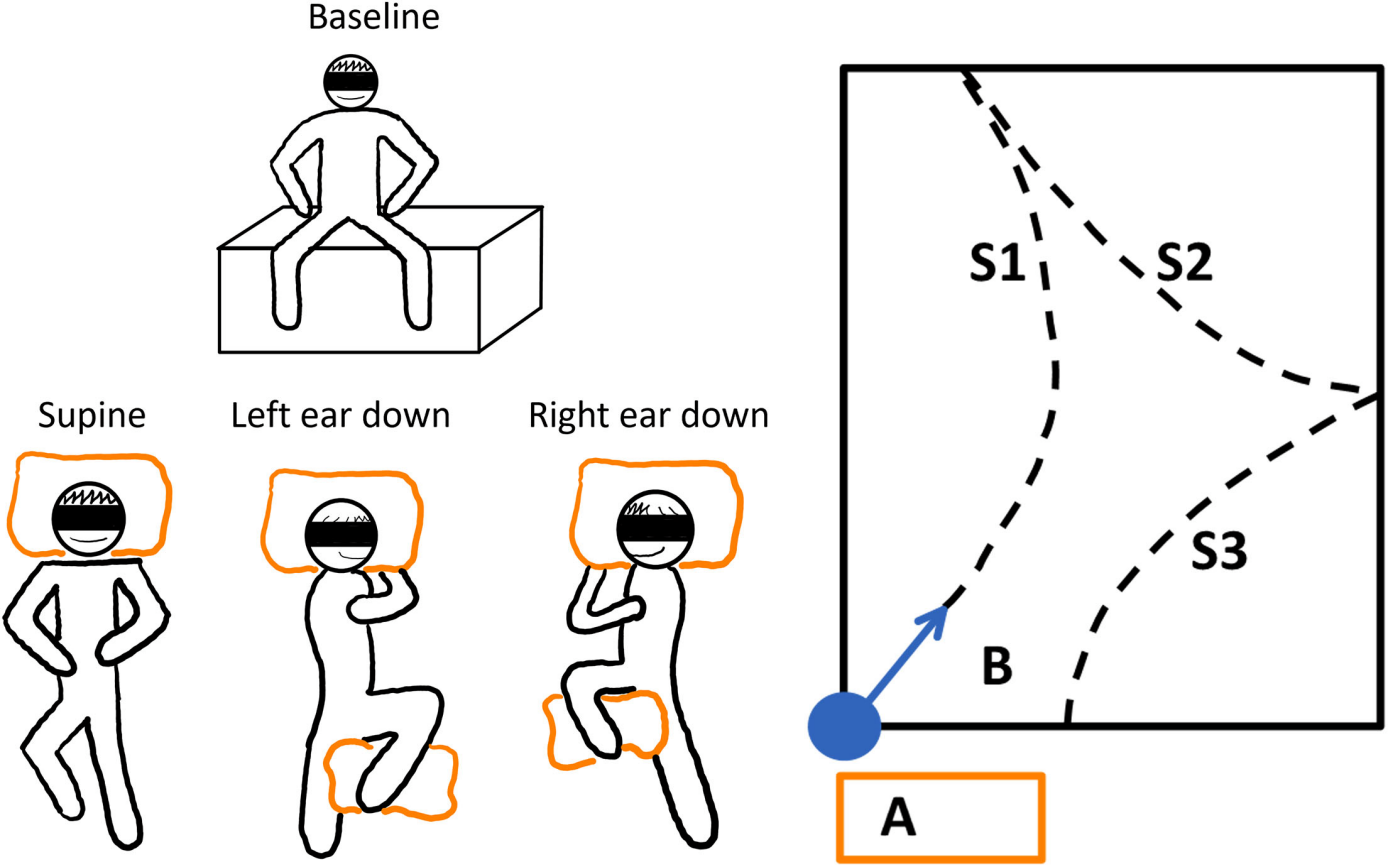
The schematic illustration of the experimental paradigm. Left panel: The baseline resting position and the three different whole-body horizontal positions are depicted. To stabilize the subjects while lying, pillows were used (orange boxes). Each trial was repeated three times. The adaptation trials were performed in randomized order after the baseline trials. Right panel: The adaptation or resting phase was conducted on vaulting boxes at position A. The subjects were guided to the starting position, and they were realigned with the sports hall's diagonal (B). The dashed lines show how a subject could have walked during the trials; WS1-3 denote the different WS.

During the adaptation phase, subjects had to rest in one of three different whole-body horizontal positions (eyes closed) for 5 min. To study the effect of gravity on perceived straight-ahead, and thus on the following walking direction, three distinct horizontal whole-body positions were defined (as illustrated in Figure 2): lying in supine position, lying on the right side (i.e., right-ear-down, RED) or lying on the left side (left-ear-down, LED). While lying, the head was positioned in reference to gravity. For the adaptation phase, a duration of 5 min was selected based on previous reports stating that adaption mechanisms during SVV adjustments occurred mostly during the first three to 5 min (7, 8, 10, 24).

At the beginning of the walking phase (before standing up from the vaulting boxes), subjects had to sit upright and slap their legs together. This event was used for later sensor-system synchronization. Next, the subjects were guided to the starting position, and they were realigned with the sports hall's diagonal. Briefly, the subjects took the neutral pose and started walking naturally and at a slow, comfortable pace. The neutral pose was defined as the subject standing upright with the feet parallel to the hips and the hands turned toward the body. The subjects were accompanied by the experimenter to allow interventions when needed. Whenever an end of the sports field was reached, the subjects were turned around (clockwise or counter-clockwise direction, direction selected randomly) and they could continue their walk. This procedure was continued until a total walking time of 2 min was reached. The ranges between the turning points are referred to as walking segments (WS). To avoid learning effects toward a straight walking direction, the subjects were guided back to the measurement starting area before removal of earmuffs and blindfolding at the end of each measurement (Figure 2, right panel).

The session started with three baseline trials in a row, which were followed by three repetitions of each of the adaptation trial conditions in random order to avoid learning effects (Figure 2, left panel).

### Data Analysis

**Figure 3:**
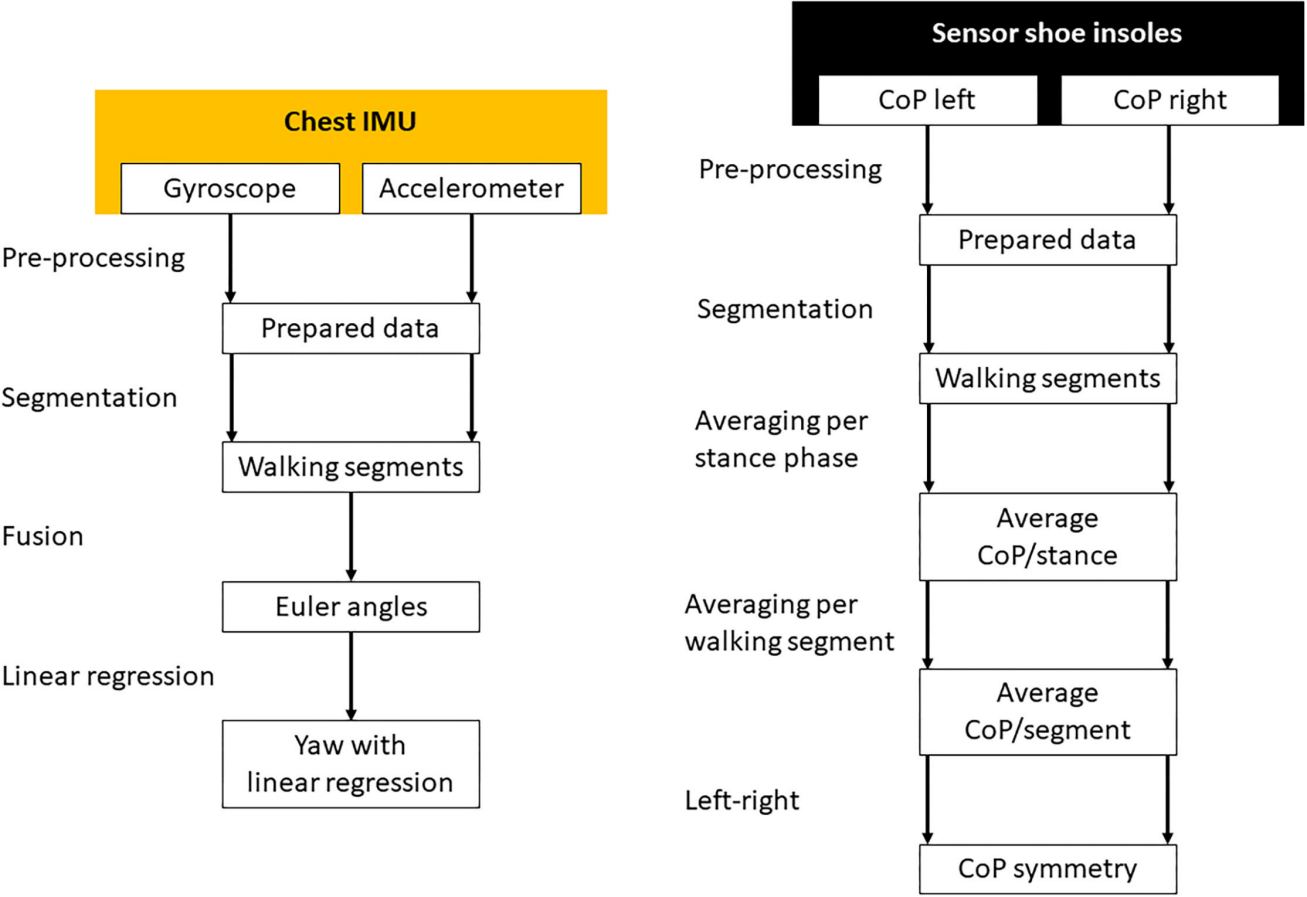
The signal processing of the chest inertial measurement unit (left) and the sensory shoe insoles (right). Missing data was interpolated and both sensor systems were synchronized during the pre-processing phase. The measurements were separated in time to different WS. Gyroscope and accelerometer data were fused to calculate Euler angles in three-dimensional space. In the resulting yaw signal, linear regressions were calculated for every WS separately. The CoP of each leg was averaged for every stance phase of each step. Afterward the CoP was averaged for every WS separately and the difference between the left and right foot calculated.

The measurements from both sensor systems were processed with MATLAB (version R2020a; The MathWorks Inc., Massachusetts, USA). A schematic overview of both sensor systems' signal processing is shown in Figure 3. The statistical analyses were performed with RStudio Version 1.4 (RStudio, Boston, USA) and R Version 4.03 (R Core Team, Vienna, Austria). Statistical comparisons were conducted with the R libraries *car* (25), *nlme* (26), *emmeans* (27) and *rstatix* (28).

#### Sensor System Synchronization

Both sensor systems were synchronized in time by detection of the leg slapping event, which was performed at the beginning of every trial. The event was detected in the signals of the left ankle motion tracker and the left sensory shoe insole. The synchronization points were identified by a threshold peak identification algorithm on the absolute medial–lateral (ML) acceleration signals.

#### Signal Scaling and Equalization

The signals of both measurement systems were scaled to standardized measurement units and equalized in time. The missing data from the measurements of both sensor systems were interpolated using modified Akima Cubic Hermite interpolation.

#### Gait–Event Detection

For the later extraction of gait–cycle dependent parameters, individual gait events (strides) were determined. Due to a timely drift between both sensor systems, the gait–event detection was performed on both systems individually. For both measurement systems, smoothed angular velocity signals of the sagittal plane were used. For the motion tracking sensors, the signals from both ankle sensors were used, for the sensory insoles, signals of the integrated IMU sensor were analyzed. Signal smoothing was obtained by a fourth-order Butterworth filter with a cut-off frequency of 12 Hz.

**Figure 4:**
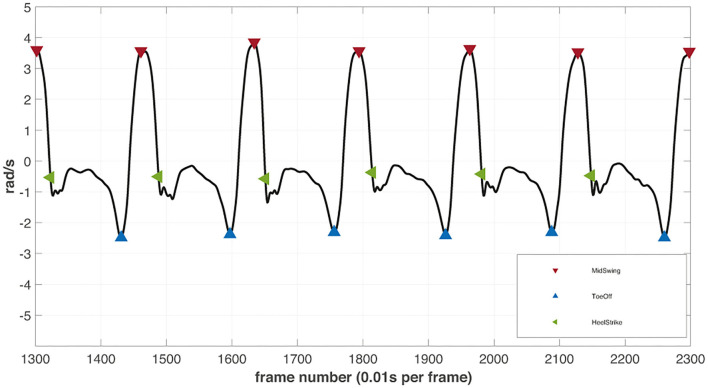
The illustrative example of filtered angular velocity signal from the right ankle inertial motion tracker with detected gait events. The detected peaks (red) in the signals are defined as mid-swing phases of every step. The lowest valley right before the mid-swing peaks are assumed to depict the toe-off events (blue). The datapoint at the zero-crossing event after every mid-swing peak determines the heel strike (green) of the next step.

As a first step, mid-swing phases were determined. It was shown that positive peaks of the angular velocity around the frontal axis denote the mid-swing phases of every step (29–31). Therefore, positive peak detection was used to determine the mid-swing phases of every gait cycle in the smoothed angular velocities around the participant's frontal axis. The peak thresholds were determined for every trial and sensor system individually. The mid-swing peaks were detected to define search windows in the signals for heel strike (following zero-crossing) and toe-off (previous valley) identification (29–32). Figure 4 shows an example of the resulting gait–event detection.

The WS were defined as phases of walking separated by passively turning subjects after reaching the border of the sports hall. The WS were determined for both sensor systems separately. The first step of every WS was found by comparing the events of both legs. The last step was defined with the same conditions but in reverse direction. Larger time differences (≥2 sec) between detected gait cycles were used to separate WS. The first and last steps were performed with high variability and thus could not be defined with an equal peak detection threshold.

#### Gait Measure Extraction

The different gait measures with the aim to describe postural control and walking direction of the participants were analyzed. Ideally, a possible trial effect would be described by both sensor systems. Two measures were calculated for every WS.

##### Linear Regression of the Yaw

The heading direction or yaw (rotation of the sagittal plane around the cranio–caudal axis) of the subjects was estimated from data of the chest motion tracker, as this sensor has shown the steadiest behavior in terms of body orientation during walking in the initial data analysis. Orientation of the chest sensor was estimated by fusing acceleration (accelerometer) and angular velocity (gyroscope) data using a Kalman filter (MATLAB function imufilter). No magnetometer data were considered. In the resulting coordinate system *x*-, *y*-, and *z*-axes were oriented in cranio–caudal, medio-lateral and PA direction respectively. Orientation was transformed to Euler angles relating to the rotations around the medio-lateral (pitch), anterior–posterior (AP, roll) and the cranio–caudal (yaw) body axes. The linear regression of the orientation of the chest around the cranio–caudal axis (yaw) was computed for each WS. The inclination of the determined regression line is further being referred to as “yaw slope.” In accordance with the IMU coordinate system, chest rotations to the left and the right were assigned positive and negative values, respectively.

##### Centre of Pressure (CoP)

The CoP sensory insole data in LM and AP was averaged for each step and each WS. To increase the separability of the results, only data recorded during the stance phase were considered.

### Statistical Investigations

For the statistical analysis of walking patterns, yaw slope and CoP in medio–lateral direction (CoP_LM_) of the first three WS were considered. As it was found that the averaged CoP_LM_ is more separable when observing the difference between the left (CoP_LM_ _left_), and the right leg (CoP_LM_ _right_), the investigations were performed based on the symmetries (CoP_LM_ _Sym_), with


(1) $$\begin{array}{r} CoP_{LM\;Sym\;}=CoP_{LM\;left\;}{-\;CoP}_{LM\;right}. \end{array}$$

Consequently, a disequilibrium to the left side would result in a negative value, to right in a positive value.

The distribution of yaw slope and CoP_LM_ _Sym_ was tested for equal variance using Bartlett's and Levene's tests and for normality in Shapiro–Wilk tests and quantile–quantile plots.

For the correlation analysis between yaw slope and CoP_LM_ _Sym_ in the first WS, the average was calculated for each trial condition for both variables and the baseline average was subtracted (baseline-corrected average data). The distribution of all the resulting data together was tested for normality using the Shapiro–Wilk test and visual histogram analysis. Correlation was analysed with the Pearson's correlation coefficient.

#### Linear Mixed Effect Models

The statistical investigation of the results was performed using linear effects models. The models were created as shown in Eq. (2) (26).


(2) $$\begin{array}{l} data.lme=lme(slope\;\sim \;trial+trial*walkingSegment,\\ random=\sim \;1\;|\;\frac{participant}{walkingSegment},\\ data=slope.data \end{array}$$

The *slope* is described by the fixed effects *trial* and *walkingSegment*, which interact with each other. The interaction was included since any effect of the adaptation phases could potentially decrease over time. Testing the model with an ANOVA showed significance in the interaction term (25, 26, 33). The main random effect *participant* was included to consider subject-dependent deviations in walking direction. The random term was nested with *walkingSegment* because any decay of a possible effect would take different lengths of time for individual subjects. The R library emmeans (27) was used to calculate estimated marginal means from the linear mixed effect models. The pairwise comparisons between trials were performed and *p*-values were calculated, using the Tukey method to adjust for the multiple testing (27).

## Results

On average, the subjects completed 4.5 ± 0.8 WS within the 2-min time limit. One subject (#3) had to repeat a single supine and one LED trial on a second examination day due to information loss during data transfer. To adjust for possible changes in the elementary drift between the test days in this subject, all three baseline attempts were repeated as well. Noteworthy, no change in the elementary drift was observed in this subject. For two subjects (#10 and #21) one RED trial was omitted for the CoP-measurements because of missing data of one foot.

### Single Subject Data for Both Sensory Systems

**Figure 5:**
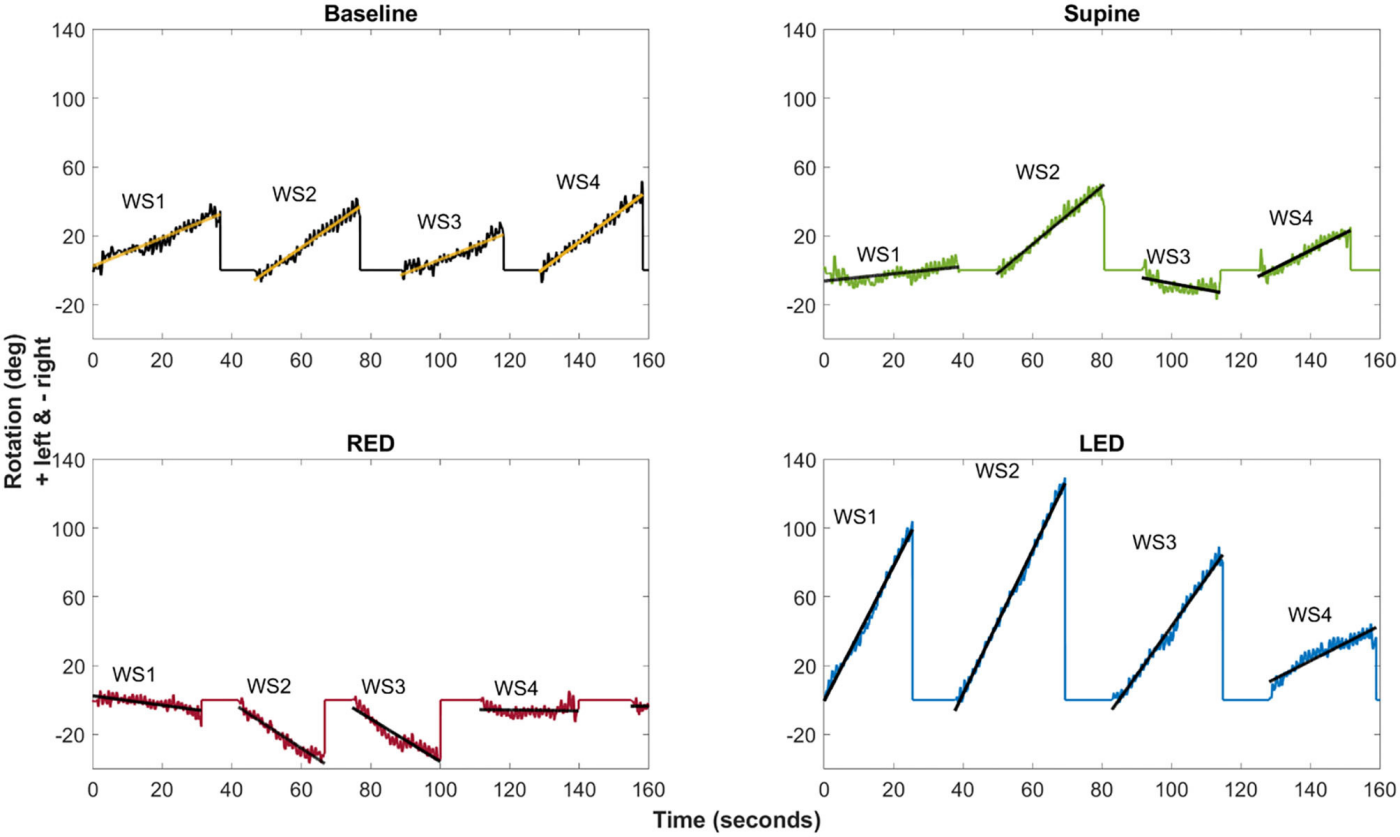
The example of the chest heading orientation (yaw) with linear regression lines for all four resting positions of a single subject (#25). The oscillations in the yaw result from upper body twists while walking. Linear regression of the yaw was computed for each WS (WS1–WS4) separately. The slope of the fitted linear regression lines (“yaw slope”) was used in statistical investigations as an estimate of heading direction. Upper left: Baseline trial showing tendency to walk to the left side. Upper right: deviation decrease to the left side during the supine trial. Bottom left: Turn to the right-hand side during RED trials. Bottom right: Turn to the left-hand side during LED trials.

The calculated chest heading orientations (“yaw”) from the IMUs with fitted linear regressions are shown in Figure 5 for all four resting positions studied for a single participant (#25). For the baseline trial, the subject showed a tendency to walk to the left side (Figure 5, upper left). In this example, the deviation to the left side decreased during the supine trial (Figure 5, upper right). The subject turned more to the right-hand side during RED trials and more to the left-hand side during LED trials (Figure 5, bottom left and right). Comparing the WS during the LED trial shows that the slope decreases for subsequent WS.

**Figure 6:**
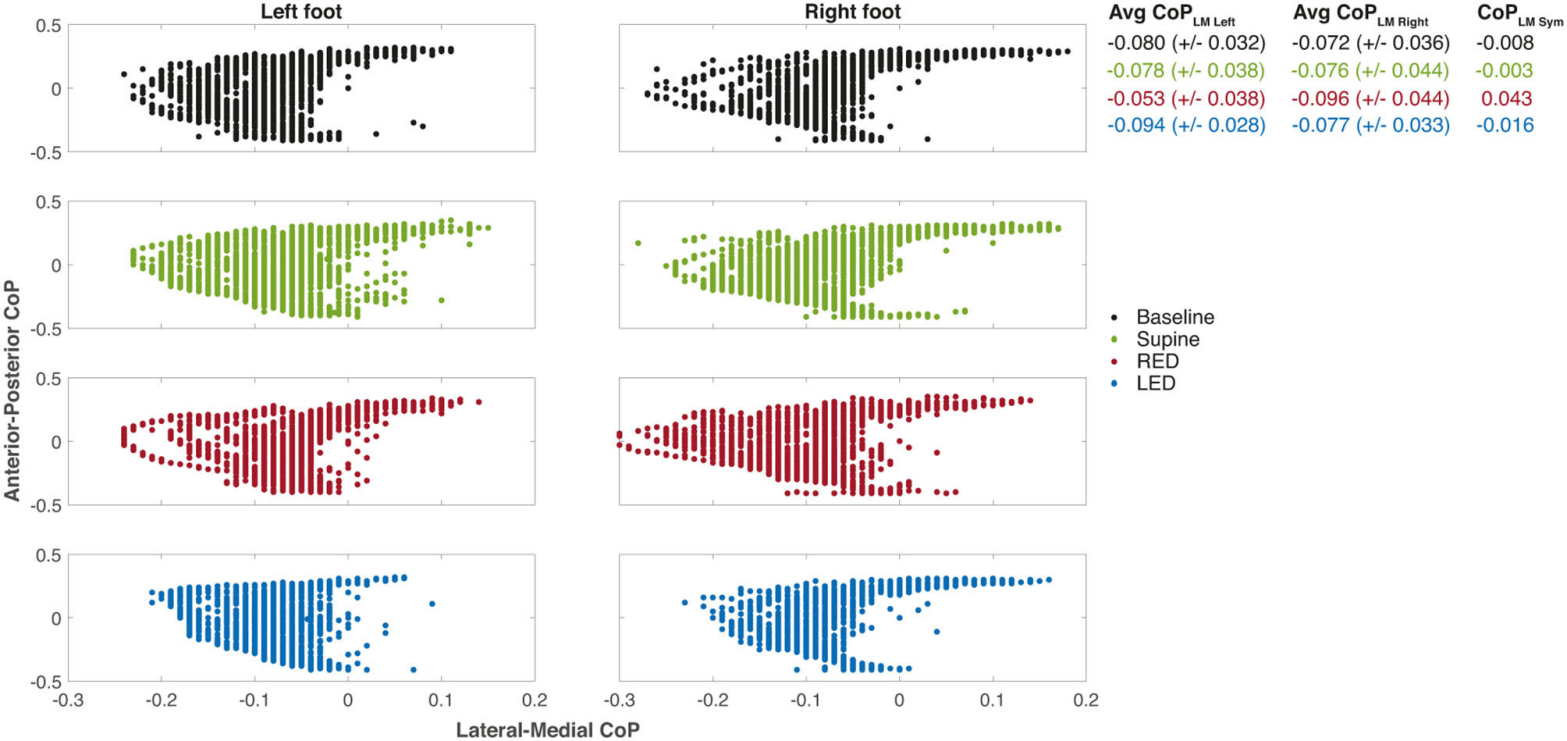
The CoP coordinates of each leg during the first WS for each resting position of the same subject. The examples depict the same measurements of subject #25 as shown in Figure 5. The positive x-axis values are medial, and the positive y-axis values are anterior CoP displacements. The CoP output of the sensory insoles on both axes ranges from −0.5 to 0.5 and is related to the corresponding insole length or width, respectively. Some values appeared more than once which is not illustrated in the figure. Averaged CoP_LM_ values are presented next to each plot for left and right foot as well as well as the CoP_LMSym_ all together with their standard deviations.

Using the sensory shoe inlets, the CoP-coordinates of each leg during the first segment are shown in Figure 6. The subject and trials are the same as shown for chest heading orientation (Figure 5). The CoP in this example generally spreads on a larger area for the right foot indicated as well as by a higher standard deviation of the average CoP_LM_. The anterior and medial tail (toe-off) is more pronounced in the right foot. Generally, the medial borders are sharper on the right foot. The LM distribution is highest during the RED trial for the right foot. Observations of the left foot show that the dispersion is lowest during the LED trials. Average CoPs show a very small difference between left and right foot during baseline and supine positions resulting in very small CoP_LM_ _Sym_ values. During RED and LED conditions the Avg CoP values become more negative for the right and left foot respectively indicating a weight shift to the lateral side. Average values for the foot opposite to the ear-down direction remain the same compared to baseline for the left foot (RED condition) and shows a slight weight shift to the left for the right foot (LED condition). CoP_LM_ _Sym_ values indicate this shift by a positive value for the RED condition and a negative value for the LED condition.

### Inter-Individual Variability in Walking Performance

**Figure 7:**
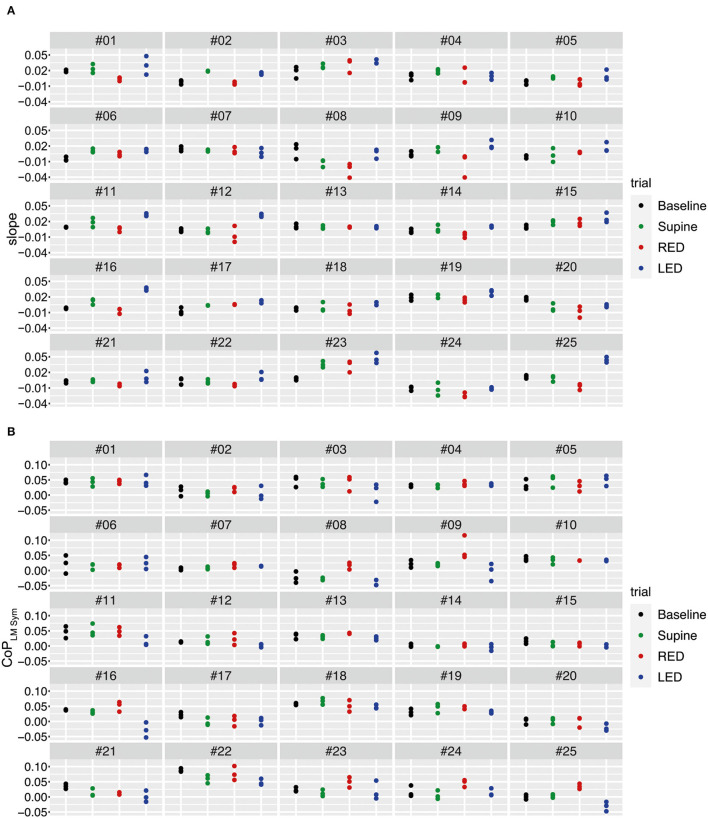
The yaw slope (slope of the linear regression of the chest heading orientation) **(A)** and differences of the averaged LMCoP_LM Sym_ (left–right) **(B)** per trial for the first WS for all 25 subjects. For **(A)**, positive slope values correspond to a rotation to the left. For **(B)**, positive differences of LM–CoP symmetries to the baseline indicate a pressure shift to the right, negative ones to the left.

The yaw slopes derived from the IMU sensors of every participant and trial for the first WS are shown in Figure 7A. During the baseline trials, the investigated population generally deviated to the left side when walking blindfolded, resulting in positive yaw slope values. The yaw slopes are mostly more positive for supine than for baseline trials. Even if the extent of the deviations varies between patients, the LED and RED conditions led in most cases to a deviation to the left and right side, respectively.

Using the sensory shoe inlets, the differences between the averaged LM–CoP symmetries are shown in Figure 7B. Absolute values for the LM–CoP symmetries differ between subjects and are though subject specific. Averaged LM–CoP symmetries (left–right foot) of the baseline and supine trials seem to be similar. During the RED condition the weight was more shifted to the right, during the LED condition more to the left compared to the baseline. The general tendencies of changes in LM–CoP symmetry between the conditions are opposite to the yaw slopes as depicted in Figure 7A, which means that the direction of the deviation is identical when comparing to the baseline data.

### Effect of Adaptation Position on Walking Direction

**Figure 8:**
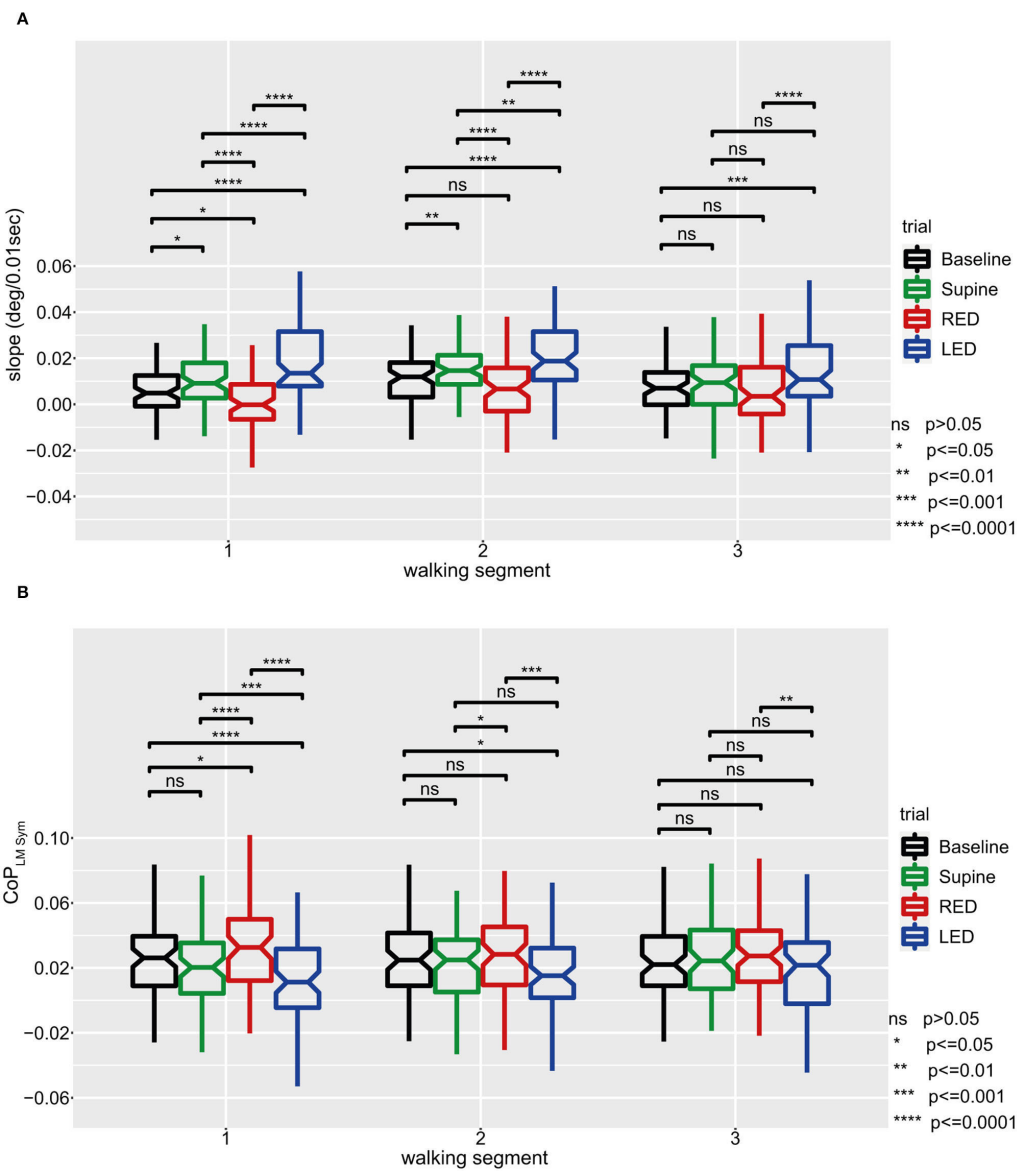
The yaw slope **(A)** and averaged LM–CoP symmetries (CoP_LM Sym_, left to right foot) **(B)** and for all subjects and trials with pairwise comparisons between trials for the first three WS. For **(A)**, positive slopes indicate yaw rotations to the left side, negative slopes to the right side. For **(B)**, the results were calculated by subtracting the averaged CoP of the right foot from those of the left foot. Baseline values are hardware related and subject specific and should not be interpreted but used as a reference value. Positive changes between two measurement conditions or WS represent a shift of weight to the right side, negative changes to the left side. For both panels, linear mixed-effects models were calculated for every WS in which all trials were compared pairwise. Comparisons and *p*-value adjustments according to Tukey are displayed on top of the boxplots according to the legend on the lower right. Boxplot details: Lower and upper edges of the boxes depict the boundaries between the 25th and the 75th percentile of the data. The lines inside the boxes represent the median for every trial. Each whisker has at most a length of 1.5 times the interquartile range (box length). Data points outside of this limit are marked as separate points. Extreme points (i.e., further apart than 3*IQR) were classified as outliers and disregarded for the analysis. An inlet (placed right to the figure) provides the level of significance by using different numbers of * symbols.

In a next step, we compared individual walking patterns and focused on the impact of the previous adaptation position. Bartlett's and Levene's tests showed that the yaw slopes do not have equal variance between the trials while CoP_LM_ _Sym_ is distributed with equal variance. Shapiro–Wilk tests and quantile–quantile plots showed that both measures are normally distributed except for the RED trials. In Figure 8A results are depicted as boxplots per trial and WS for the IMU sensors. The general deviation of the investigated population to the left, in form of a positive shift, can also be observed in this figure. For the first WS, all trials significantly differ from each other. The deviation to the left side was larger for the supine laying trials compared to baseline trials and was highest when subjects rested LED. The only distribution with a median heading rotation toward the right side can be observed for the RED trials. The difference between the trials was largest when comparing yaw slopes from RED trials with those from LED trials.

The boxplots and significance values show decreasing differences between the trials for subsequent WS. In the third WS, results significantly differed only between the LED trials and the baseline trials, and between the LED trials and the RED trials.

In Figure 8B results are depicted as boxplots per trial and WS for the sensory shoe inlets. The distributions of the difference between both feet of the averaged LM–CoP symmetry show that, except for the baseline compared to the supine trials, all trials significantly differ to each other for the first WS. Compared to the baseline, a shift in the positive direction can be observed for the RED trials and in the negative direction for the LED trials. The distribution difference between the trials is highest when comparing RED and LED trials. The difference between the trials decreases for subsequent WS. In the third WS, results only significantly differ between the RED and LED trials. For the baseline and supine trials, no significant difference was found for any WS.

**Figure 9:**
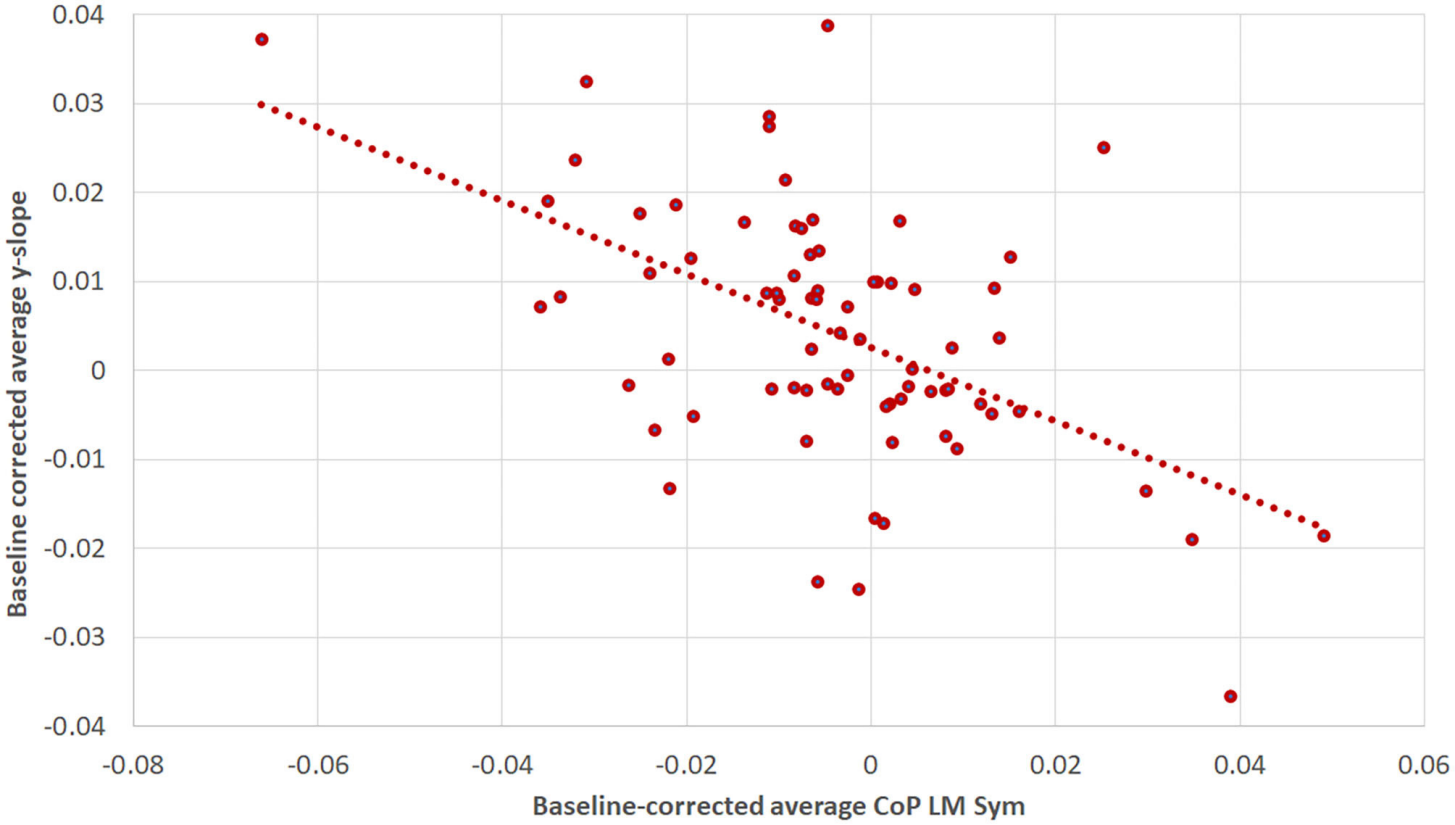
The correlation between baseline-corrected average yaw slope and baseline-corrected average CoP_LM Sym_ for the first WS. The Pearson correlation coefficient was R = −0.518 (*p* < 0.001). The figure is composed of three data points per subject: one for the supine, one for the RED and one for the LED condition.

The correlation analysis of the baseline corrected and averaged yaw slope and CoPLM Sym per trial condition during the first walking revealed a moderate negative correlation (*R* = −0.518; *p* < 0.001) (34), as shown in Figure 9.

## Discussion

Asking healthy human subjects to walk straight-ahead blindfolded after prolonged whole-body roll-tilt to either side, we observed a significant bias in post-adaptation walking in darkness into the direction of the previous roll-tilt position. This bias was observed in two independent measurement systems for gait tracking and decreased again over the time.

### An Overall Leftward Bias in Perceived Straight-Ahead

Yaw data showed a tendency to deviate toward the left side while walking blindfolded both in the baseline trials and in the “neutral” adaptation trials (i.e., in supine position) in the majority of subjects. This leftward bias was significantly stronger for the supine adaptation condition than the baseline condition in the first two WS as recorded by the IMU motion tracking system, while this difference disappeared again in the third WS, possibly indicating a decay in adaptational mechanisms due to prolonged supine roll position. This overall leftward tendency could also be depicted on the adaptation trials with preceding RED or LED resting position. While subjects deviated significantly more toward the left after adaptation in LED position, the overall walking direction was still slightly leftward after adaptation in RED position.

The reason for this overall leftward bias when walking blindfolded remains unclear. Previously, lateralized cortical processing of vestibular input has been reported. Perception of straight-ahead was studied in patients with unilateral vestibular deficits (UVD), indicating side-specific deviations in subjective straight-ahead (SSA). While patients with left-sided UVD demonstrated a contralesional shift in SSA, the SSA remained accurate in patients with right-sided UVD (35). Thus, the authors concluded that their data support the hypothesis of an asymmetric vestibular function in healthy human subjects. Others have demonstrated an overall right-hemispheric dominance of the vestibular cortex as well (36, 37). With regards to the observed overall tendency to deviate toward the left side in the post-adaptation trials, this could be linked to such asymmetric representation of higher cortical vestibular properties. Lack of reporting of such an effect in the literature on blindfolded walking could be related to the total walking distance, being much shorter (5–8 m) in previous publications, the specific experimental paradigms applied or the patient populations studied.

When investigating larger walking distances when blindfolded, healthy human subjects tend to walk in sometimes surprisingly small circles (<20 m diameter), though rarely in a systematic direction (38). The authors proposed accumulating noise in all components of the sensory system to explain such non-systematic deviations from straight-ahead. Noteworthy, no correlations between the laterality of these deviations and functional asymmetries (handedness/footedness) were found. Thus, on top of a systematic post-tilt bias, accumulating noise may have affected the precision of walking in our study. Noteworthy, the duration of walking blindfolded was much longer in the study by Souman and colleagues (38) than in the work presented here, possibly triggering different walking patterns over larger time scales.

### The Pathophysiology of the Observed Post-Tilt Walking Bias

Five minutes of the whole-body static roll-tilt to either side were sufficient to induce significant shifts in walking direction compared to lying on the back during the adaptation period. Over the time, the effect of the adaptation in ear-down position on walking direction decreased, indicating partial re-adaptation to the current (non-biased) condition. Thus, over a period of 2 min walking a decrease, but not complete cessation of the adaptation effect can be noted. While in the current paradigm no decay time constant of the post-tilt bias can be calculated, the pattern observed suggests a decay time constant in the range of a few minutes. Noteworthy, using the pressure measurement sensory shoe insoles, observed differences in walking direction in the adaptation trials disappeared faster, but were still significant for RED *vs*. LED post-adaptation trials in WS 3.

We have previously discussed the pathophysiological bases for such a post-tilt bias on the SVV (10, 14) and SHV (14). In brief, we have proposed a shift in the internal estimate of direction of gravity based on recent experience (prior knowledge) in the context of Bayesian optimal observer theory (see introduction). As a result, the perceived direction of gravity will be shifted toward the body-longitudinal axis, thus immediately after returning back upright shows a shift toward the previous static whole-body roll-tilted position. We predicted that a shift in perceived earth–vertical in the roll-plane affects walking direction as well, which was confirmed in our study demonstrating a clear bias in perceived straight-ahead toward the previous adaptation position.

A potential mechanism to explain an effect of prolonged static roll-tilt on translational responses could be related to the continuous otolith stimulation while lying on the side. Thus, in left/right ear-down position gravity pulls down the otoliths of the utricles and activates them continuously. This stimulus for the receptors is identical to an ongoing linear translational acceleration. Consequently, an adaptive process to this acceleration could occur that is maintained for a certain amount of time after returning to the upright position. In this position, the adaptive response would be reoriented in a latero-lateral adaptation, that, in turn, would influence the SSA and thus translatory motor activity. While this mechanism emphasizes adaptational effects of body resting position on central coordinates, alternatively, prolonged whole-body roll tilt could act on the motor system of gait by changing directly one side otolithic descending motor output. Thus, the walking directional shift observed in our study cannot be directly related with a shift of the SSA. However, taking into account previous studies emphasizing adaptational mechanisms on both perceived direction of gravity (10, 14) and self-motion perception (19, 20) by prolonged (asymmetric) otolith stimulation, such a direct mechanism seems less likely.

The path integration in both healthy human subjects and patients with either acute or chronic vestibular deficits has been extensively studied [see (39) for review]. Previously, the galvanic vestibular stimulation has been applied to study the role of the vestibular organs in path integration (40), demonstrating significantly increased arrival errors and angular errors in a virtual triangle completion task. Likewise, the repetitive transcranial magnetic stimulation (rTMS) has been used to disrupt path integration in a vestibular navigation task, demonstrating deteriorated perceived contralateral spatial displacement after rTMS in the area of the right posterior parietal cortex (41).

In patients with UVD, veering has been noted in non-visually guided walking tasks (42, 43), with walking trajectories deviating toward the lesioned side when walking straight-ahead over short distances (5.5 m) (17) and significantly larger final arrival errors in a triangular-path walking task have been reported (44).

### Comparison of Two Independent Gait Analysis Systems

Whereas we measured trunk yaw rotation in the first setup, distribution of pressure on the feet was assessed in the second setup. Both systems detected significant deviations in heading while walking blindfolded toward the side of the adaptation position during the first WS in the post-adaptation trials, with results from both setups correlating significantly. Thus, our findings were confirmed using two independent gait measurement systems that monitored distinct parameters of heading simultaneously. Specifically, when biasing internal estimates of perceived straight-ahead, walking patterns in participants were modified in such a way that both the difference in distribution of pressure between the left and the right foot and the yaw rotation of the trunk were pointing toward the adapted side.

However, when assessing the temporal evolvement of the deviations over the whole walking distance, the dynamics were distinct. Overall, the level of significance was higher when comparing the different post-adaptation test conditions and more frequently found also in the 3^rd^ WS when assessing the body-fixed accelerometers/gyroscopes compared to the pressure sensors placed in the participant's shoes. Thus, the first system was superior in detecting deviations in walking direction for the paradigm studied here. These discrepancies are most likely related to the selection of the parameters to be monitored, the signal-to-noise ratio of the sensors implemented and inter-individually varying walking habits, foot shape and type of shoes used. Based on these observations, with the proposed parameters, the use of trunk-fixed gyroscopes/accelerometers currently seems more suitable to detect deviations in walking direction accurately and precisely in future studies than pressure sensors located in shoe inlets. On the other side, the handling of the inlets was less demanding, thus selection of devices for recordings depends also on the requirements (temporal/spatial resolution, signal-to-noise ratio) of the specific paradigm to be implemented.

### Clinical Implications of a Heading Bias

While none of our healthy young participants showed signs of truncal instability or even demonstrated (near) falls, this post-tilt bias may become clinically relevant in the elderly and in patients with pre-existing gait disorders. Taking into account the sleeping habits, lying on the side at night may increase the risk of falls and fall-related injuries when getting up in the morning or during the night, especially if vision is impaired. Furthermore, besides building up a directional bias during a good night's sleep, internal estimates may also be less precise in the morning. It has been demonstrated that immediately after getting up in the morning the trial-to-trial variability of perceived earth–vertical is significantly larger than in the evening (45). Thus, special attention should be paid in the morning when getting up to minimize the risk of falls and fall-related injuries.

### Limitations

The size of the indoor walking hall requiring splitting up the entire walking over 2 min into several segments with experimenter-guided rotations of the participant's orientation before continuing walking, resulting in potential clues while repositioning. Importantly, we controlled for direction of rotation-specific effects between WS and did not find any significant differences. Thus, we do not think that this segmented walking significantly biased our findings.

Deviations from straight-ahead while walking were determined indirectly from parameters indicating uneven pressure distribution (shoe soles) or yaw trunk rotation. We have chosen this strategy as due to growing offsets over time of the sensory input obtained from both systems, performing a path integration was not feasible. Nonetheless, using two independent measurement systems, comparable results in heading patterns after whole-body roll-tilt adaptation could be obtained, strongly supporting the existence of such a post-tilt bias.

## Conclusions

In summary, a significant bias in perceived straight-ahead after static whole-body roll-tilt in either left-ear-down or right-ear-down position over periods as short as 5 min further confirms the impact of prior knowledge on spatial orientation and navigation. Specifically, it emphasizes the broad impact of a shifting internal estimate of direction of gravity over a range of distinct paradigms, illustrating similar decay time constants of this bias. For tracking changes in walking direction, a set of body-fixed accelerometers/gyroscopes seems superior to foot pressure measurements when considering averaged data, which should be taken into account for future studies. The observed bias in perceived straight-ahead likely has also implications in daily life and especially in the context of fall-prevention. It emphasizes that getting up in the morning after a good night's sleep is a vulnerable period, with an increased risk of falls and fall-related injuries due to not optimally tuned internal estimates of direction of gravity and direction of straight-ahead.

## Data Availability Statement

The raw data supporting the conclusions of this article will be made available by the authors, without undue reservation.

## Ethics Statement

The studies involving human participants were reviewed and approved by Ethikkommission Nordwest- und Zentralschweiz (EKNZ, ID=2020-01712). The patients/participants provided their written informed consent to participate in this study.

## Author Contributions

AT: conception of the work, interpretation of data for the work, drafting the work, and revising it critically for important intellectual content. VD: acquisition, analysis and interpretation of data for the work, drafting the work, and revising it critically for important intellectual content. DB: interpretation of data for the work and revising the work critically for important intellectual content. SH: conception of the work, interpretation of data for the work, and revising the work critically for important intellectual content. All authors approved the final version of the manuscript and agreed to be accountable for all aspects of the work in ensuring that questions related to the accuracy or integrity of any part of the work are appropriately investigated and resolved. The authors confirm that all persons designated as authors qualify for authorship, and all those who qualify for authorship are listed.

## Funding

VD was financially supported by the Cantonal Hospital of Baden (Grant #009192).

## Conflict of Interest

The authors declare that the research was conducted in the absence of any commercial or financial relationships that could be construed as a potential conflict of interest.

## Publisher's Note

All claims expressed in this article are solely those of the authors and do not necessarily represent those of their affiliated organizations, or those of the publisher, the editors and the reviewers. Any product that may be evaluated in this article, or claim that may be made by its manufacturer, is not guaranteed or endorsed by the publisher.
